# Diagnosis of Perforated Appendiceal Diverticulitis Following Laparoscopic Appendectomy

**DOI:** 10.7759/cureus.83939

**Published:** 2025-05-12

**Authors:** Paulo Sousa, Eduarda Magalhães, Marta Marques, Joana Cracel Lourenço, Joaquim M Costa Pereira

**Affiliations:** 1 General Surgery, Unidade Local de Saúde de Braga, Braga, PRT; 2 Colorectal Surgery, Unidade Local de Saúde de Braga, Braga, PRT

**Keywords:** acute appendicitis, diverticulitis, emergency surgery, gastrointestinal surgery, laparoscopic surgery

## Abstract

Appendiceal diverticulitis is a rare and often underdiagnosed condition that poses significant diagnostic challenges due to its atypical clinical presentation and similarity to acute appendicitis. We describe the case of a 37-year-old female patient who presented with acute abdominal pain and was ultimately diagnosed with appendicitis through computed tomography (CT). The patient underwent a successful laparoscopic appendectomy. This case underscores the diagnostic complexity of appendiceal diverticulitis, which is frequently mistaken for other causes of acute abdomen. The condition may be congenital or acquired, with differing pathophysiological mechanisms and histological features. Given the risk of complications such as perforation or neoplasia, prophylactic appendectomy is recommended even in cases of non-inflamed diverticula. Histopathological examination remains essential for definitive diagnosis. This case highlights the importance of heightened clinical suspicion and individualized surgical management in improving patient outcomes.

## Introduction

Appendiceal diverticulitis, although rare, has gained increasing recognition in the medical literature. First described by Kelynack in 1893 [1], appendiceal diverticulosis remains an uncommon condition, with reported incidence ranging from 0.004% to 2.1% [2].

As a distinct form of appendiceal pathology, it poses notable diagnostic challenges due to its atypical presentation. Clinical symptoms may vary widely, ranging from acute abdominal pain to subtle, nonspecific signs that can mimic other abdominal conditions. Its presentation often overlaps with that of acute appendicitis, the most common appendiceal disease, but appendiceal diverticulitis carries a fourfold higher risk of perforation and may also be associated with underlying neoplasms [3].

Numerous case reports and series emphasize the importance of maintaining high clinical suspicion, given the variability in presentation. Imaging modalities such as computed tomography (CT) and ultrasound have significantly improved diagnostic accuracy [4]. In addition, several studies have explored the association between diverticulitis and appendiceal tumors, underscoring the importance of thorough evaluation.

Accurate differentiation from acute appendicitis is critical, particularly in view of differing prognoses and management strategies. While conservative treatment has been proposed in select cases, surgical intervention remains the mainstay in most instances. Ongoing research into the pathogenesis and molecular mechanisms of appendiceal diverticulitis continues to enhance our understanding of this rare condition.

This case report adds to current knowledge by summarizing key findings and providing further insights into diagnostic and therapeutic considerations, potentially guiding future research in this evolving field.

## Case presentation

We present a case report of a patient admitted to our hospital's emergency department. The clinical records were reviewed, and demographic details such as age and gender were collected. Additionally, clinical features including abdominal pain, nausea, anorexia, and diarrhea were analyzed. Diagnostic evaluation comprised inflammatory markers and imaging studies. Intraoperative findings were also assessed in detail.

A 37-year-old female patient, independent in daily activities and living with her husband and children, presented to the emergency department with diffuse abdominal pain of one day's duration, predominantly in the periumbilical region. On a subsequent visit, she reported persistent pain in the right lower quadrant and periumbilical area, accompanied by fever and nausea. Symptoms had evolved over the previous week, although she denied any changes in bowel habits. Her medical history included peripheral venous disease and hypertension. Past surgeries involved tubal ligation and excision of a skin cyst. Regular medications included oral contraceptives and trazodone.

On physical examination, the patient was alert, oriented, and in good general condition. She exhibited normal respiratory function but was mildly hypotensive with associated tachycardia and fever. Abdominal inspection was unremarkable; bowel sounds were present, but palpation revealed tenderness in the left lower quadrant and epigastrium, with rebound tenderness and guarding.

Laboratory results showed neutrophilic leukocytosis and elevated inflammatory markers, as illustrated in Table 1.

**Table 1:** Laboratory findings upon admission Comprehensive laboratory data of the patient upon arrival at the emergency department, including complete blood count, biochemical parameters, coagulation profile, liver and pancreatic enzymes, and electrolytes. All values are presented alongside their respective reference ranges. Abnormal findings include elevated leukocytes, neutrophilia, and increased C-reactive protein.

Parameter	Patient values	Reference range
Complete blood count		
Erythrocytes	4.38 ×10⁶/μL	3.8 – 5.0 ×10⁶/μL
Hemoglobin	12.6 g/dL	11.9 – 15.6 g/dL
Hematocrit	36.9 %	36.6 – 45.0 %
Mean corpuscularc volume (MCV)	84.2 fL	82.9 – 98.0 fL
Mean corpuscular hemoglobin (MCH)	28.8 pg	27.0 – 32.3 pg
Mean corpuscular hemoglobin concentration (MCHC)	34.1 g/dL	31.8 – 34.7 g/dL
Red cell distribution width (*RDW*)	11.8 %	11.7 – 14.4 %
Leukocytes	14.1 ×10³/μL	4.0 – 11.0 ×10³/μL
Neutrophils	81.2 % (7.5 ×10³/μL)	45 – 75 % (1.8–7.7 ×10³/μL)
Eosinophils	0.8 % (0.08 ×10³/μL)	0 – 0.5 %
Basophils	0.3 % (0.03 ×10³/μL)	0 – 0.1 %
Lymphocytes	14.6 % (1.4 ×10³/μL)	12 – 34 %
Monocytes	3.1 % (0.3 ×10³/μL)	2 – 9 %
Platelets	240 ×10³/μL	150 – 400 ×10³/μL
Coagulation pofile		
Prothrombin Time (PT)	13.3 sec	8.0 – 14.0 sec
International normalised ratio (*INR*)	1.14	0.8 – 1.2
Activated partial thromboplastin time (aPTT)	31.9 sec	25.0 – 37.0 sec
Biochemistry		
Glucose	129 mg/dL	74 – 106 mg/dL
C-reactive protein (CRP)	167.6 mg/L	< 5.0 mg/L
Urea	24 mg/dL	19 – 49 mg/dL
Creatinine	0.8 mg/dL	0.60 – 1.20 mg/dL
Electrolytes		
Sodium	139 mmol/L	136 – 145 mmol/L
Potassium	3.8 mmol/L	3.5 – 5.1 mmol/L
Chloride	104 mmol/L	98 – 107 mmol/L
Magnesium	1.02 mg/dL	1.3 – 2.1 mg/dL (geral)
Liver and pancreatic enzymes		
Aspartate aminotransferase (AST, glutamic-oxaloacetic transaminase (GOT))	19 U/L	12 – 40 U/L
Alanine transaminase (ALT, glutamic pyruvic transaminase (GPT))	19 U/L	7 – 40 U/L
Alkaline phosphatase	93 U/L	45 – 116 U/L
Amylase	54 U/L	30-118 U/L
Lipase	26 U/L	13 – 60 U/L
Lactate dehydrogenase (LDH)	161 U/L	120 – 246 U/L
Creatine kinase (CK) total	104 U/L	34 – 145 U/L
Myoglobin	26	< 110 ng/mL
Bilirubin (total)	1.02 mg/dL	0.3 - 1.2 mg/dL
Bilirubin (direct)	0.33 mg/dL	< 0.3 mg/dL

**Figure 1:**
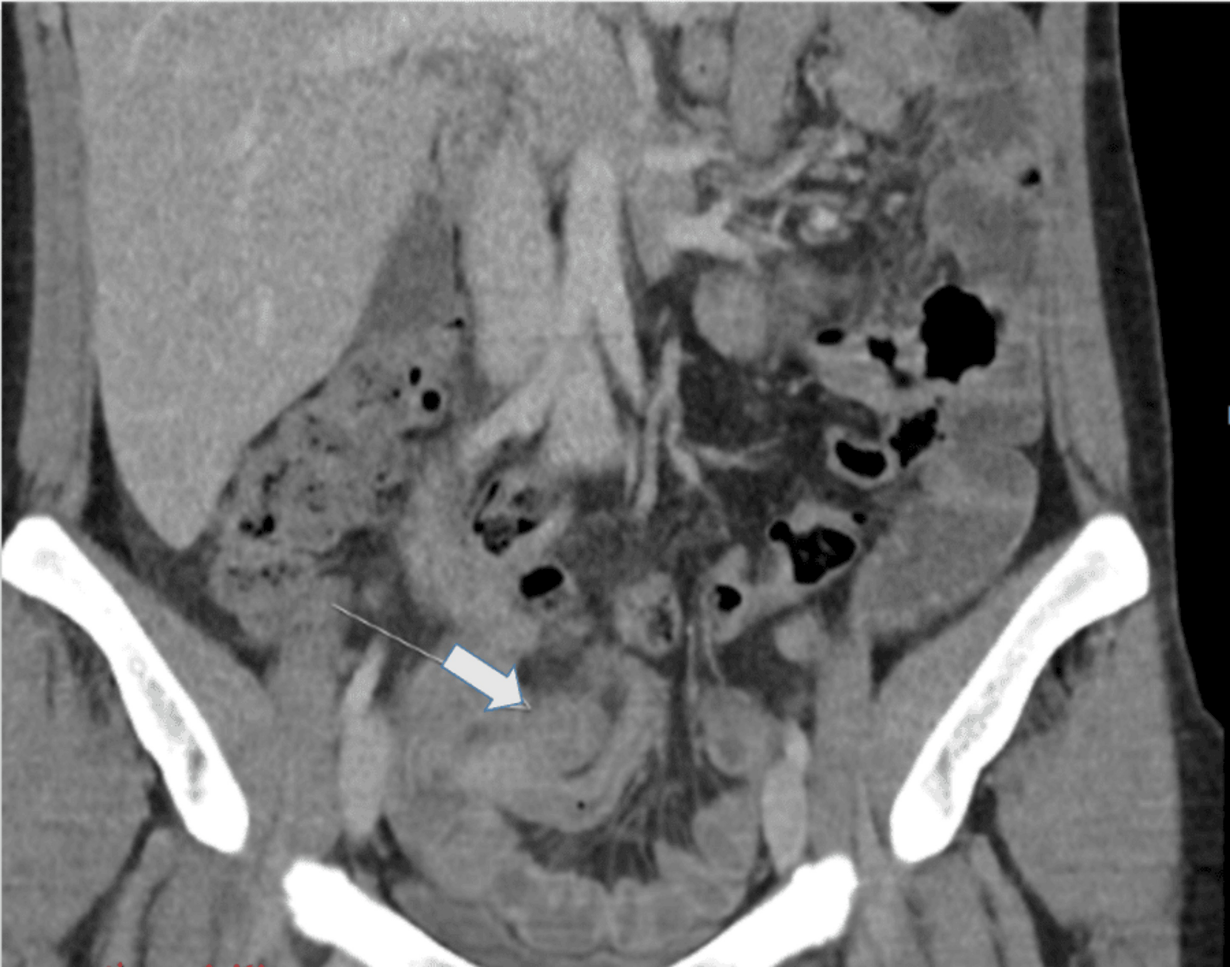
Computed tomography (CT) revealing signs of appendicitis (white arrow) Contrast-enhanced axial CT image showing an enlarged tubular structure arising from the lateral aspect of the cecum with a glove-finger appearance, consistent with an inflamed appendix. An adjacent hypodense area measuring approximately 10 × 25 mm suggests a small phlegmon or localized collection.

Abdominal CT demonstrated an enlarged intestinal loop originating from the lateral aspect of the cecum, extending to the hypogastric region with a “glove-finger” appearance near the midline, suggestive of an inflamed appendix/acute appendicitis. A nearby hypodense area (approximately 10 × 25 mm) was compatible with a small phlegmon or organized collection. Thickening of the adjacent terminal ileum suggested inflammatory involvement. The appendix was in contact with the sigmoid colon, which showed mild parietal thickening. Mesenteric lymphadenopathy and a small amount of free fluid in the Douglas pouch were noted. The liver, gallbladder, biliary tree, pancreas, kidneys, and adrenal glands were unremarkable. The spleen was at the upper limit of normal size (12.8 cm) with homogeneous texture. The uterus appeared globular and deviated to the right; both adnexa were free of masses. The bladder was nearly empty, with no significant wall or intraluminal abnormalities, as shown in Figure 1.

The proposed treatment was laparoscopic appendectomy following clinical stabilization, analgesia, and initiation of empirical antibiotic therapy. Despite generalized peritonitis and a phlegmon near the cecum, the surgical approach remained a standard laparoscopic appendectomy, as the pathology was deemed manageable via a minimally invasive technique. Intraoperatively, extensive inflammatory adhesions involving the uterus, cecum, and sigmoid colon were noted. No intra-abdominal abscesses were identified beyond the localized phlegmon, and a drainage was placed. The patient’s postoperative course was uneventful, with resolution of symptoms and no complications observed during the one-month outpatient follow-up.

**Figure 2:**
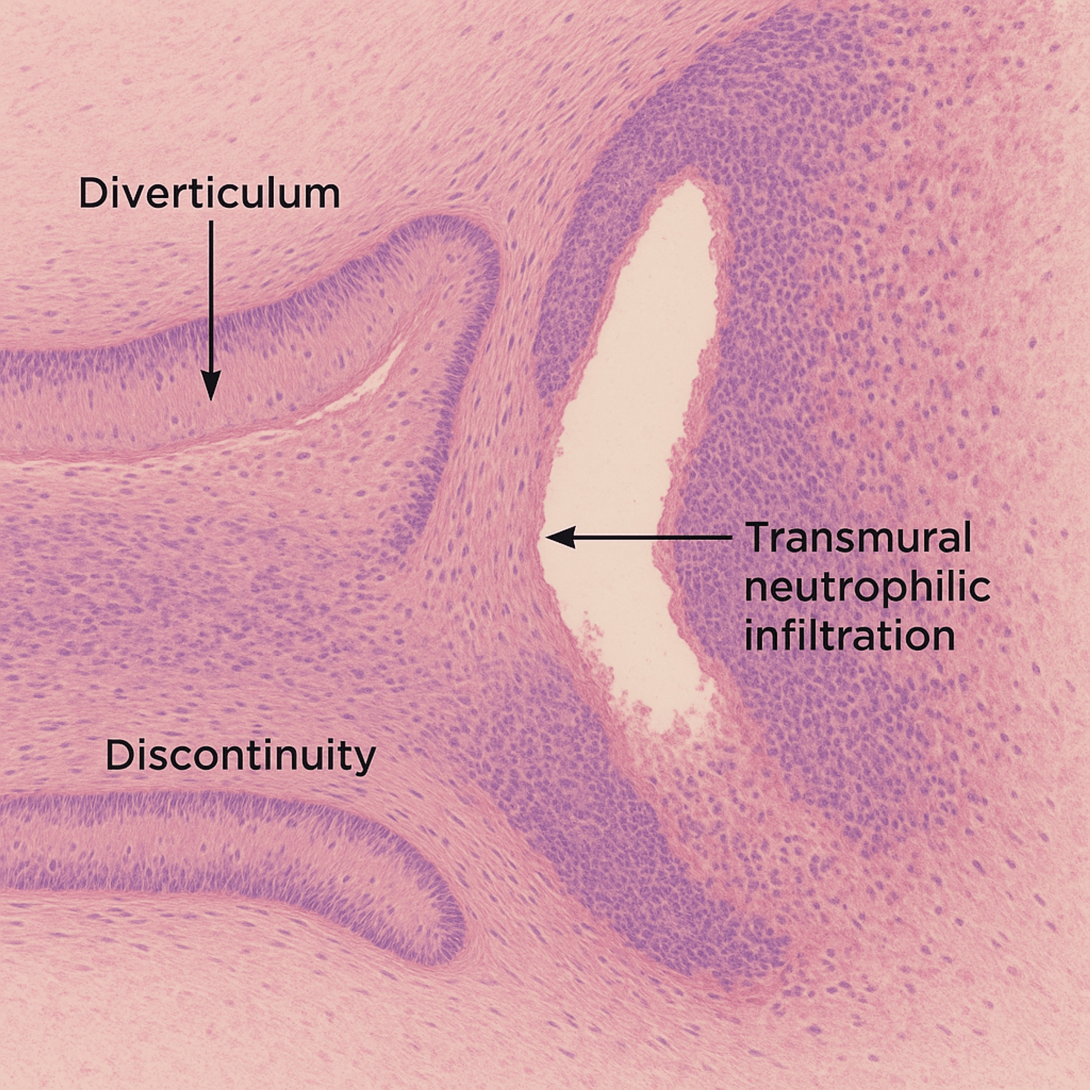
Histological section of perforated appendiceal diverticulitis Mucosal diverticulum with wall discontinuity and transmural neutrophilic infiltration extending to the serosa, consistent with acute perforated diverticulitis. Adjacent mucosa remains unremarkable.

Histopathological analysis of the 7 cm appendix revealed congested serosa with fibrinous exudate and false membranes. The appendix showed irregular caliber (1.5 cm) with an obliterated lumen. A 1 × 0.8 cm mucosal diverticulum was identified, presenting a 0.7 cm perforation in the distal third. Microscopically, a perforated diverticulum with granulocytic infiltration extending to the serosa was observed. The surrounding mucosa showed no significant alterations. The final diagnosis was perforated diverticular disease of the ileocecal appendix with secondary peritonitis, as illustrated in Figure 2.

## Discussion

This case highlights the diagnostic complexity of appendiceal diverticular disease, which may be misinterpreted as acute appendicitis, particularly when presenting as advanced peritonitis secondary to a perforated diverticulum.

There are two distinct types of appendiceal diverticula, each with its own incidence: congenital (0.014%) and acquired (1.9%) [3]. Acquired diverticula arise from increased intraluminal pressure, resulting in mucosal outpouching through the muscularis propria at weak points along the mesenteric and antimesenteric borders. Contributing factors may include fecaliths, proximal tumors, and excessive mucus production [5]. Conversely, congenital diverticula involve outpouching of all three layers of the appendix wall, are typically located on the antimesenteric border, and are occasionally associated with genetic syndromes such as Patau syndrome [6].

In 1971, Luc Deschênes et al. proposed a five-type morphological classification system [7], which includes primary diverticulitis; acute diverticulitis secondary to acute appendicitis; a non-inflamed, simple diverticulum identified on histological analysis; a simple diverticulum with acute appendicitis (in which the diverticulum itself is uninvolved); and chronic peri-diverticulitis associated with acute appendicitis. In 1989, Lipton et al. suggested a similar classification comprising four types: acute diverticulitis with a normal appendix; acute diverticulitis with acute appendicitis; non-inflamed diverticulum with appendicitis; and non-inflamed diverticulum with a normal appendix. Types I-III are further subclassified according to the presence or absence of perforation, with Type I being the most common, accounting for 40% to 50% of cases [8].

Although clinical and surgical management in the acute phase may not differ significantly between complicated appendicitis and diverticulitis of the appendix, the latter may present radiological features such as focal wall outpouching, and it is histologically distinct. Moreover, appendiceal diverticulitis requires increased histological scrutiny due to its stronger association with neoplastic transformation. Several studies, including Dupre et al., have shown an association between appendiceal diverticulosis and neoplastic lesions in up to 48% of cases, particularly mucinous neoplasms [5]. This contrasts with colonic diverticulosis, which is not typically linked to an increased risk of malignancy.

This case underscores the diagnostic overlap between appendiceal diverticulitis and complicated appendicitis. The final diagnosis was only confirmed histologically, reinforcing the necessity for thorough pathological assessment of all appendectomy specimens.

## Conclusions

Appendiceal diverticulitis may mimic acute appendicitis and is often only recognized postoperatively. While surgical management remains similar, histopathological analysis is crucial for definitive diagnosis and identification of potential neoplastic risk. This case highlights the need for increased clinical suspicion and routine pathological examination in cases of suspected appendicitis.
